# Prevalence of elongation and calcification patterns of elongated styloid process in south India

**DOI:** 10.4317/jced.50981

**Published:** 2013-02-01

**Authors:** R Sudhakara Reddy, Ch Sai Kiran, N. Sai Madhavi, M N. Raghavendra, A. Satish

**Affiliations:** 1 Professor and HOD. Department of Oral Medicine and Radiology, Vishnu Dental college, Bhimavaram; 2 Postgraduate. Department of Oral Medicine and Radiology, Vishnu Dental College, Bhimavaram; 3 Senior Lecturer, Department of oral medicine and radiology,Vishnu Dental College, Bhimavaram

## Abstract

Objective: Very few studies have been reported in the literature classifying the elongation and calcification patterns of styloid process. The present study was done to investigate the prevalence of elongation and calcification patterns of styloid process in patients attending a dental institution in south India. Study design: 600 digital panoramic radiographs of patients with dental problems were obtained from the outpatient department of the Dental institution. The apparent length of the styloid process was measured by a single experienced oral radiologist, with the help of the measuring tools on the accompanying software. The type of elongation and calcification patterns of each elongated styloid process was classified as per Langlai’s classification with few modifications. Finally the data was subjected to statistical analysis. Results: Out of 520 measurable styloid processes (260 panoramic radiographs), 154 styloid processes had length greater than 3cm. The mean average length of elongated styloid process was 3.67±0.62 cm. No significant association was obtained between age and length of styloid process. In present study, the type of elongation pattern has no effect on the calcification pattern. Our results suggested that Type I elongated styloid processes were most likely to be completely calcified (type D), but statistically not significant. Conclusion: Type I pattern of elongation was found to be more prevalent in elder age group and was completely calcified in most of the cases. Interestingly, only three patients (out of 260) showed symptomatic elongation of styloid process. A relatively high prevalence of type IV elongation pattern (9 /154) was obtained in the present study, when compared to type III styloid process. Further large scale imaging studies are required to evaluate the presence of type IV elongation pattern in various population groups.

** Key words:**Eagle’s syndrome, Elongated styloid process, Stylopharyngeous muscle, Tonsillectomy.

## Introduction

Styloid process is a long slender and pointed bony process projecting downwards, forwards, and slightly medi-ally from the temporal bone. It developed from Reichert’s cartilage of 2nd branchial arch. Eagle an Otorhinolar-yngologist, first described elongated styloid process, also known as Eagle’s syndrome in 1937 (1). The normal length of styloid process is approximately 20-30 mm (2). The styloid process length which is longer than 30 mm was considered to be elongated styloid process (1,2). Studies in India have estimated that in 19.4 – 52.1% of the general population there was radiographic evidence of an elongated styloid process, the highest (52.1%) being recorded in the region of Mathura (north India) (3). Elongated styloid process presents with symptoms that may include a dull, aching pain localized in the throat, with or without referred pain to the ear and mastoid region on the affected side. Some patients may complain of pain on swallowing (dysphasia) or an abnormal sensation of a foreign body in the pharynx. But most cases are asymptomatic. Eagle’s syndrome is diagnosed by both radiographical and physical examination. More commonly, a panoramic radiograph is used to determine the styloid process elongation. Although there are many suggested hypotheses, the exact etiology of calcified and ossified elongated styloid process is unknown.

There were few studies reported on elongation and calcification patterns of styloid process. Authors like Lang-lais RP (4), MacDonald-Jankowski (5), Correll (6) have classified these patterns of elongated styloid process. In the present study, our aim was to investigate the prevalence of elongation and calcification patterns of elongated styloid process in patients attending our institution using digital panoramic radiographs.

## Material and Methods

**Figure 1:**
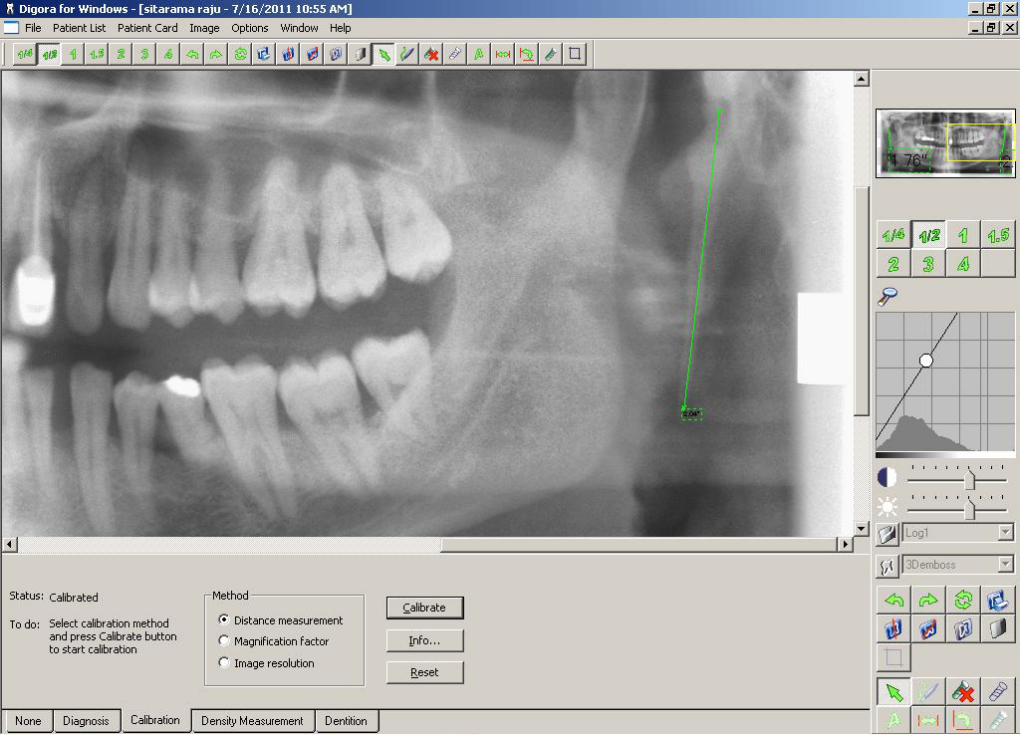
Digital measurement of length of elongated styloid process using Digora software.

After obtaining permission from the ethical committee, 600 digital panoramic radiographs of patients with dental problems, who were enrolled in the department during 2010-2011, were obtained. The panoramic radiographs of 340 patients who have questionable styloid process and having position and magnification errors were excluded. Only 260 Panoramic radiographs (520 styloid processes) showed measurable styloid process. These radiographs were obtained from panoramic system (X-MIND PANOCEPH, Sorodex, Finland.) using Photo-Stimulable-Phosphor (PSP) sensors under standard exposure factors, as recommended by the manufacturer. The apparent length of the styloid process was measured by a single experienced oral radiologist, with the help of the measuring tools on the accompanying software (Digora for Windows 2.7.103.437 network client, copyright © 1993-2010 Sorodex), (Fig. 1). The magnification factor used for the machine was 1.26. The length of styloid process was measured in a similar method, described by Ilgüy et al (2), as the distance from the point where the styloid left the tympanic plate to the tip of the process, regardless of whether or not the styloid process was segmented. The styloid process measuring more than 30mm was considered as elongated (2). The type of elongation and calcification of each styloid process on both right and left sides were classified as per Langlais et al (4), with few modifications. In our study we found few radiographs showing calcification of styloid process which were not continuous with the base of the skull. Similar type of presentation was reported by MacDonald – Jankowski DS (5), in his classification. Hence we modified Langlai’s classification by adding a 4th variant of elongation pattern. It mainly included styloid process similar to type “H to J” patterns (proposed by MacDonald – Jankowski DS) of calcified stylohyoid chain which was not continuous with the base of skull.

**Figure 2:**
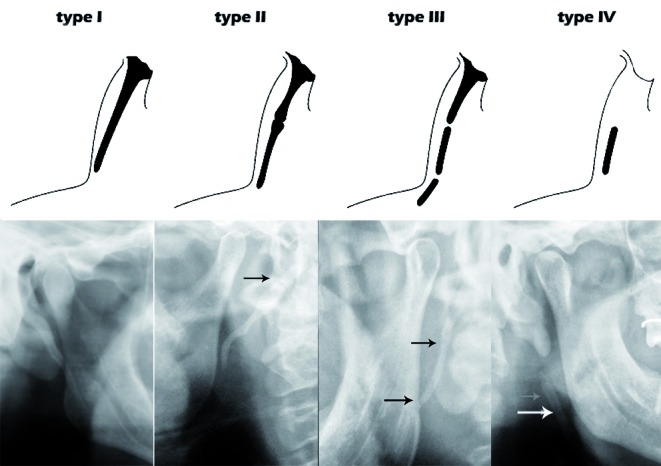
Schematic and radiographic representation of elongation patterns of styloid process.

**Figure 3:**
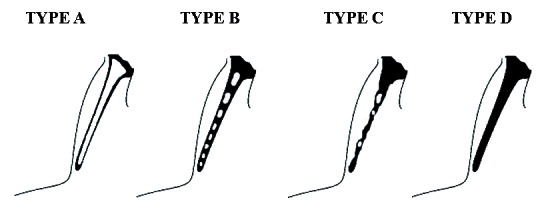
Schematic representation of calcification patterns of styloid process.

The elongation patterns (Fig. 2) were graded as type I (Uninterrupted integrity of styloid process (>30mm)), type II (Styloid process joined to the mineralized stylomandibular or stylohyoid ligament by a single pseudoarticulation), type III (segmented styloid process containing multiple pseudo articulations) and type IV (elongation of styloid process due to distant ossification). The calcification patterns (Fig. 3) were divided into type A (styloid process showing calcified outline), type B (partially calcified styloid process with discontinuous radiolucent core), type C (nodular appearance of styloid process with varying degree of central radiolucency) and type D (completely calcified styloid process with no evidence of a radiolucent interior).

The collected data was entered in a spreadsheet (Excel 2007, Microsoft office) and was analyzed using, statistical analysis software (SPSS version 16.01, SPSS.inc, Chicago, 1989-2007). Spearman’s rank correlation test, t-test and chi square test were used to determine any significant differences between the groups.

## Results

Out of 520 styloid processes 154 styloid processes (95 males and 59 females) measured more than 3cm (elon-gated). Of them 125 styloid processes (81%) showed Type I elongation pattern and 90 styloid processes (58%) showed Type D calcification patterns. The mean length of styloid process in various age groups was represented in  Table 1.

**Table 1:**
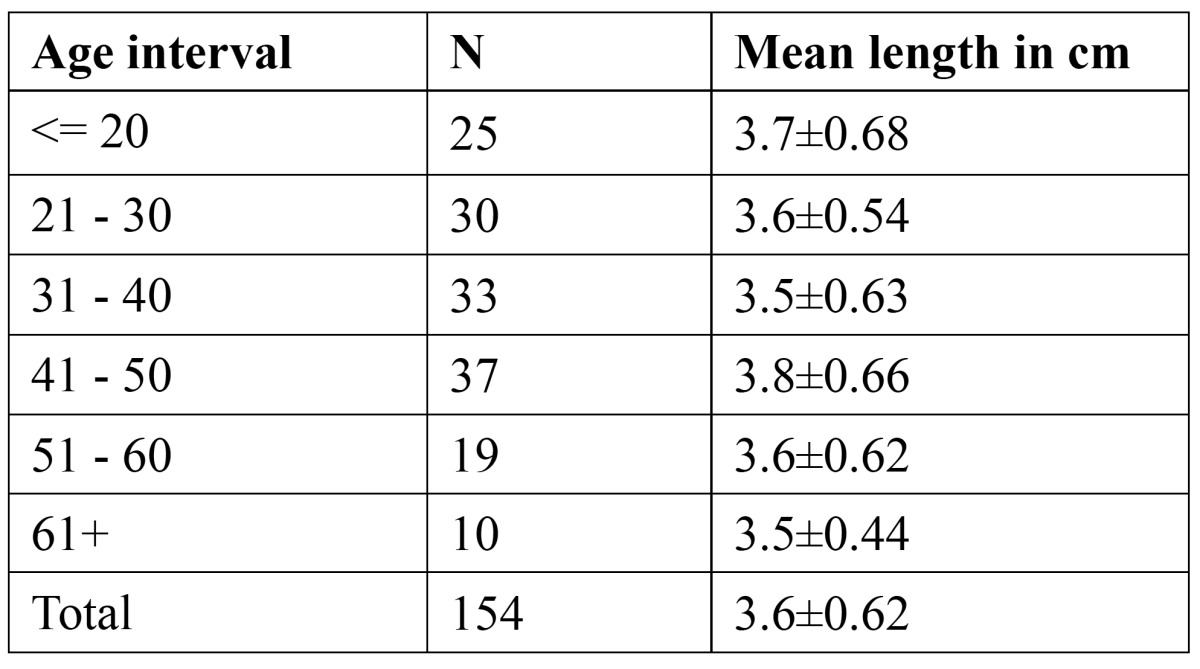
Mean length of styloid process in various age groups.

- Effect of gender on elongated styloid process:

The mean length of elongated styloid process was 3.67±0.62 cm. An independent sample T test was conducted to compare the mean length of elongated styloid process in males and females. The mean styloid process length was higher in male (3.70±0.68 cm) than female (3.58±0.52 cm) patients, but no statistically significant difference was obtained.

- Effect of age on styloid process elongation:

The mean age of patients with elongated styloid process was 37.95±14.58 years. The mean age of patients with elongated styloid process in males and females was 39.36±14.47 and 35.69±14.61 years respectively. There were no statistically significant differences in mean age of males and females (p >0.05).

Spearman’s rank correlation was computed to assess the relation between age and length of styloid process. The correlation was almost found to be zero between age and length and was not statistically significant (p>0.05).

- Effect of age on elongation and calcification patterns:

The types of styloid process elongation and the pattern of calcification stratified according to age intervals were shown in Table 2 and  Table 3, respectively. Type I (elongated) was the most frequent type of styloid process (125/154). The most frequent patterns of calcification were completely calcified (type D) (90/154). No significant association was observed between age – elongation patterns and age - calcification patterns respectively (P>0.05).

**Table 2:**
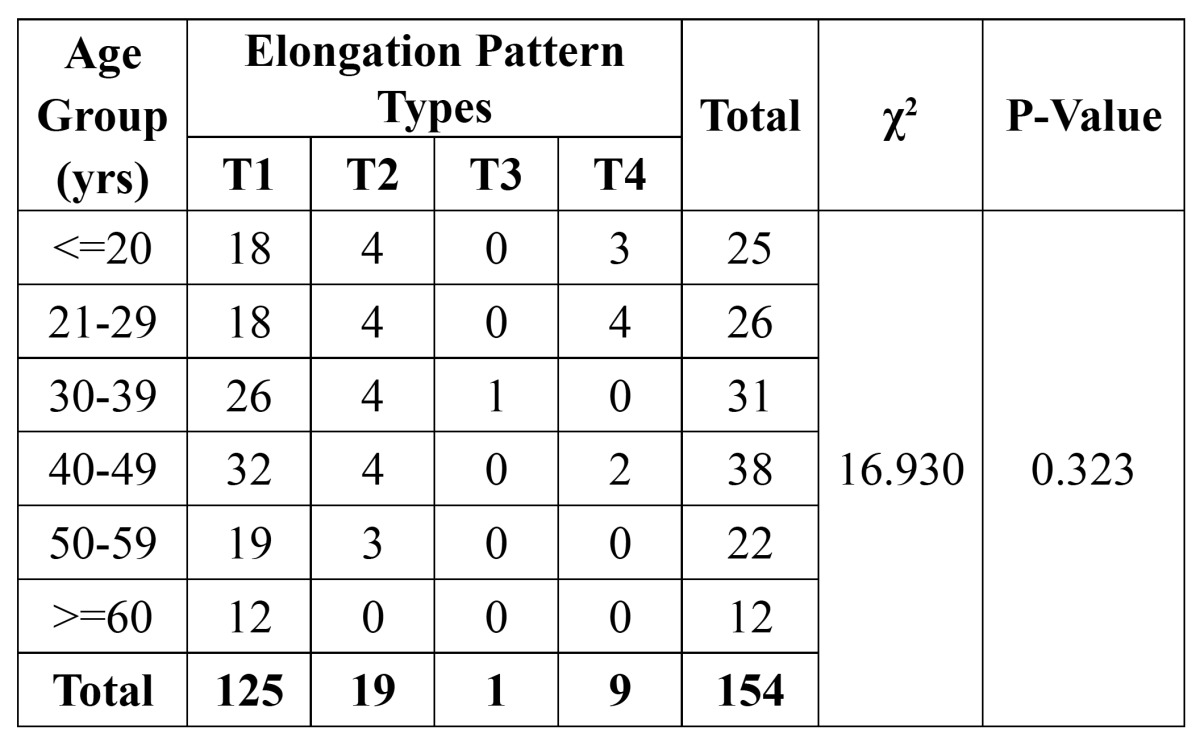
Sample distribution according to age group and elongation pattern.

**Table 3:**
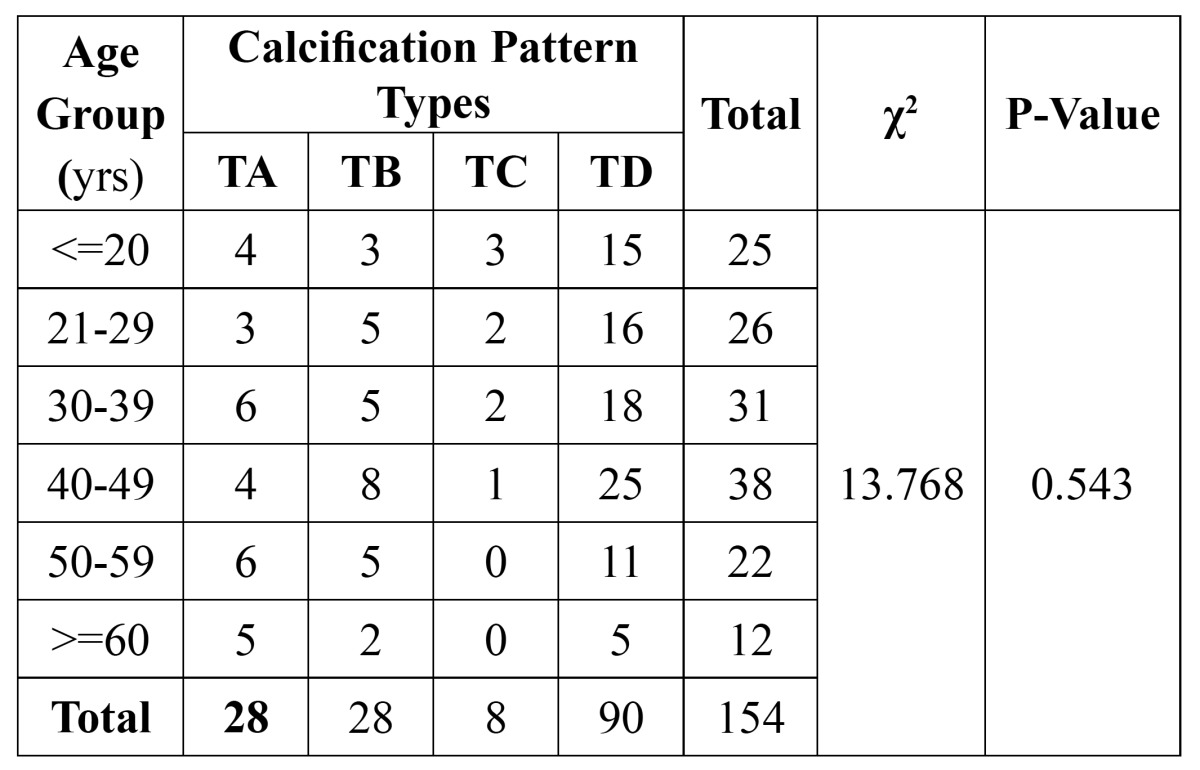
Sample distribution according to age group and calcification pattern.

- Effect of gender on elongation and calcification patterns:

Type I (elongated) was the most frequent type of styloid process in both males (78/95 [82%]), and females (47/59 [80%]). Only 1 patient (male) was classified as having type III segmented elongated styloid process measuring 426 mm long and the patient was asymptomatic. The most frequent pattern of calcification was completely calcified (type D) in both males (53/95 [56%]) and females (37/59 [63%]). No significant association was observed between gender - calcification patterns and elongation patterns (P>0.05).

- Effect of elongation patterns on calcification patterns:

The association between elongation and calcification patterns was shown in  Table 4. The results showed that most of the Type I elongated styloid process (55.2%) were completely calcified (Type D) .The association between calcification and elongation patterns was not statistically significant (P>0.05).

**Table 4:**
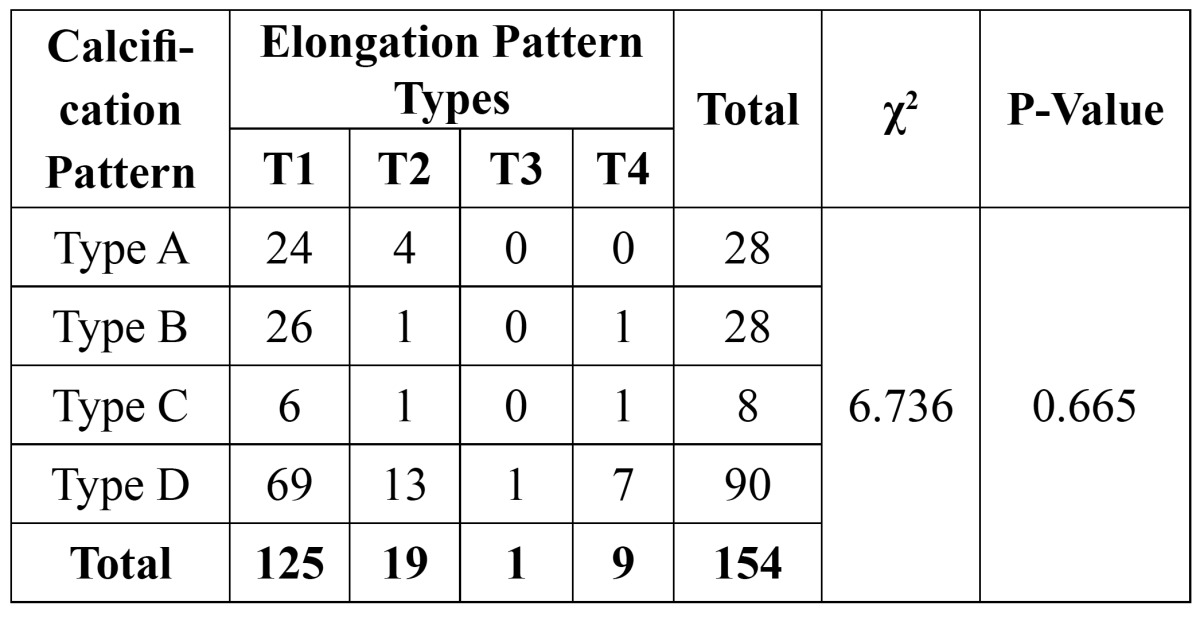
Calcification and Elongation pattern in the study sample.

## Discussion

Variation is the law of nature. Every human is unique anatomically to such an extent that even identical twins are not alike. The term “styloid process” is derived from the Greek word ‘Stylos’ meaning a pillar. The styloid process is normally a cylindrical bone which arises from the temporal bone in front of the stylomastoid foramen. The attached structures include stylopharyngeous, stylohyoid and styloglossus muscles and stylohyoid and stylomandibular ligaments.

The entire stylohyoid apparatus is developed from 4 segments.

1. Tympanohyal portion- base of the styloid process.

2. Stylohyal portion –shaft of styloid process 

3. Ceratohyal portion –stylohyoid ligament

4. Hypohyal portion – lesser cornu of hyoid bone.

The mechanism of ossification of these parts is not fully understood. It is suggested that the stylohyoid ligament retains some part of cartilage within during ossification which resulted in varying degrees of ossification and elongation of stylohyoid chain. ‘Elongated styloid process’, a term used since a publication by Eagle in reports concerning findings in dentomaxillofacial and ear- nose- throat patients (1). Eagle’s definition was that the normal styloid process measures between 2.5 to 3cms. His method of measurement was not described but his examples showed lateral radiographs of the skull. Anatomical variation in the length of the styloid process and its stylohyoid chain is of profound anatomical, anthropological as well as clinical importance. There are many variations of styloid chain, including the thickness of segments, angle and direction of deviation, length of process and degree of calcification. It is necessary for defining the type of elongation and calcification of each styloid in order to describe its radiographic appearance.

Hence in order to simplify the description, Langlais et al. (4) had classified styloid process based on the type of elongation and calcification. Langlai’s classification of elongated styloid process is based on three types of complexes—Type I, elongated; Type II, pseudoarticulated; and Type III, segmented. These types are further described by a pattern of calcification: calcified outline, partially calcified, nodular, and completely calcified (5). But in our study we found few cases (9/154) which showed absence of a continuous calcified styloid process from the base of skull i.e calcifications involving portions of Stylohyal, Ceratohyal or Hypohyal portions. Hence we modified Langlai’s classification by adding a 4th variant of elongation pattern. It is similar to type “H to J” pattern of calcified stylohyoid chain classified by MacDonald – Jankowski DS (5).

In our study, as in Erol (8), we used panoramic radiographs of the patients to enable us to identify any elongated styloid process. The average length of styloid process was 3.67±0.62 cm. The mean age of the patients with elongated styloid process was 37.95±14.58 years.

Elongated styloid process is frequently asymptomatic (9). Only a very small portion of patients have clinical presentation. Similarly in our study only 3 cases were reported with symptomatic elongation of styloid process. The 3 patients were over 35 years of age. It has been reported that the majority of symptomatic patients have no recent history of tonsillectomy or other cervicopharyngeal trauma and neither of our patients did (10). Out of 3 patients, one patient had bilaterally elongated styloid process, while others had a unilateral elongated styloid process. One patient had a type II elongated styloid process.

Our study mainly emphasized on the effect of age and sex on length, elongation and calcification patterns of styloid process. But there was no significant association between age – elongation patterns and calcification patterns which was in accordance to the study of Balcioglu HA et al (11). Specifically our study results suggest that elongated styloid process (type I elongation pattern) in most of the patients was completely ossified (type D calcification pattern), but not statistically significant.

A relatively high prevalence of type IV elongation pattern (9/154) when compare to type III (1/154), was ob-tained in the present study, which can be attributed to the following reasons:-

1. Hypomineralisation of styloid process in the region of base of skull resulting in the loss of structural appea

ance in the panoramic radiograph.

2. Increased density of the soft tissue structure (ear lobe) masking the presence of a portion of radiopaque styloid

process, in the radiograph.

3. Presence of carotid artery calcifications in the region of styloid process simulating the presence of type IV

styloid process (12).

4. Artefactual presence of radiopaque structures in the region of styloid process masking its presence.

Hence the presence of type IV styloid process should be confirmed only by higher diagnostic imaging modalities like Computed tomography (CT) or Cone beam computed tomography (CBCT). But unfortunately, being a retrospective study, the advantage of re-analyzing these structures in our study was very minimal.

In conclusion, to the best of our knowledge, this is the first study in terms of prevalence of the styloid process elongation and calcification patterns in our region. Further studies are still required to evaluate the presence of Type IV styloid process and also to investigate the relation between the type of styloid process and symptomatic presentation (Eagle’s syndrome) in patients.
